# A decline of LAMP- 2 predicts ursodeoxycholic acid response in primary biliary cirrhosis

**DOI:** 10.1038/srep09772

**Published:** 2015-04-20

**Authors:** Lu Wang, Guan-ya Guo, Jing-bo Wang, Xin-min Zhou, Qiong Yang, Zhe-yi Han, Qiang Li, Jing-wen Zhang, Yun Cai, Xiao-li Ren, Xia Zhou, Rui-rui Chen, Yong-quan Shi, Ying Han, Dai-ming Fan

**Affiliations:** 1 Division of Hepatology, Xijing Hospital of Digestive Diseases, The Fourth Military Medical University, Xian, 710032, Shaanxi Province, China; 2 Department of Digestive Disease, 153 Central Hospital of PLA, Zhengzhou 450042, Henan Province, China

## Abstract

Biochemical response to ursodeoxycholic acid (UDCA) in patients with primary biliary cirrhosis (PBC) is variable. We have previously reported that augmented expression of lysosome-associated membrane protein 2 (LAMP-2) was correlated with the severity of PBC. This study aimed to determine whether serum LAMP-2 could serve as a predictor of biochemical response to UDCA. The efficiency of serum LAMP-2 to predict biochemical response was assessed after 1 year of UDCA treatment in PBC patients by a retrospective analysis. We found that the basal serum LAMP-2 level was increased in PBC, especially in patients with stage III-IV (*p* = 0.010) or TBIL > 1 mg/dL (*p* = 0.014). Baseline serum LAMP-2 was higher in non-responders than that in responders, but the difference was statistically insignificant. However, after UDCA treatment, serum LAMP-2 level decreased prominently in the first 3 months, which was more obvious in responders. Further studies showed that the 35% decline of LAMP-2 after treatment for 3 months could be stated as an indicator of UDCA response with the sensitivity of 62.9% and specificity of 75.0% by Paris criteria. Meanwhile the specificity and sensitivity were identified as 63.5% and 64.1% by Barcelona criteria. Together, a decline in LAMP-2 might help to predict the response to UDCA.

Primary biliary cirrhosis (PBC) is a slowly progressive cholestatic disease associated with the development of cirrhosis and eventually liver failure^1^,^2^. Currently, ursodeoxycholic acid (UDCA) is the only approved therapy for PBC, resulting in the reduction of biochemical markers of cholestasis and leading to the normalization of survival in most patients^3^,^4^,^5^,^6^. However, up to one-third of patients may not achieve an adequate biochemical response, a factor independently associated with disease progression, liver transplantation and death^7^,^8^,^9^,^10^,^11^,^12^.

Identification of PBC patients with poor outcome mainly based on biochemical response is an important issue in clinical practice as well as in the design of therapeutic trials. Though the efficiency of several combinations of serum bilirubin, alkaline phosphatase (ALP), and aspartate aminotransferase (AST) threshold values was assessed to predict the long-term efficacy of UDCA^7^,^8^,^9^,^10^,^13^, there is still no common consensus on the definition of the biochemical response^13^. Hitherto, the Paris criteria were recognized as the most effective and practical one^7^,^14^.

Previously published criteria for predicting biochemical response were mainly applied after UDCA therapy for 1 or 2 years^7^,^8^,^9^,^10^. However, it is necessary to distinguish the patients suitable for alternative therapy other than UDCA treatment as soon as possible. It has been recommended that therapeutic trials should target patients with incomplete biochemical response after 3 to 6 months of UDCA treatment^14^. However, a biochemical response identified as early as 3 to 6 months after treatment was only evaluated in few large independent cohorts of PBC patients^11^,^15^.

Lysosome-associated membrane protein 2 (LAMP-2) is a heavily glycosylated type 1 membrane protein^16^. The X chromosome carries the gene encoding LAMP-2, which structurally consists of a small cytoplasmic tail with a lysosomal membrane targeting signal, a transmembrane domain, and a large intraluminal head^17^. Although LAMP-2 has been confirmed with essential roles in vasculitis^18^, adhesion^19^, and cellular homeostasis, including autophagocytosis^20^ and antigen presentation^21^, the function of LAMP-2 is still uncertain. We have previously reported that the increased expression of LAMP-2 in liver correlated with the severity of PBC, and UDCA treatment may partially improve the recovery of LAMP-2^22^, suggesting augmented expression of LAMP-2 may assist in the progress of PBC and predict a poor outcome in patients with PBC.

In the present study, our aims were: (1) to determine the basal levels of serum LAMP-2 in patients with PBC; (2) to evaluate whether the baseline serum LAMP-2 could serve as a predictor of biochemical response to UDCA; (3) to determine the predictive potency of serum LAMP-2 within the first year of UDCA treatment; (4) to evaluate whether a decline of serum LAMP-2 could prospectively distinguish patients with unsatisfactory biochemical response.

## Results

### Patient Demographics

A total of 102 PBC (76 female, 26 male) patients with a mean age of 54 years (ranged from 31 to 73) were included in the study. The demographic and clinical features of the PBC patients at the beginning and after 1 year of UDCA therapy are listed in Table 1.

Sera from the 126 subjects with viral hepatitis B (HBV), 114 subjects with viral hepatitis C (HCV), 27 subjects with intrahepatic cholestasis (IC), and 84 healthy volunteers were included as controls, the basal biochemical characteristics of which was presented in Table 2.

### Elevation of baseline serum levels of LAMP-2 in patients with PBC

**Figure 1:**
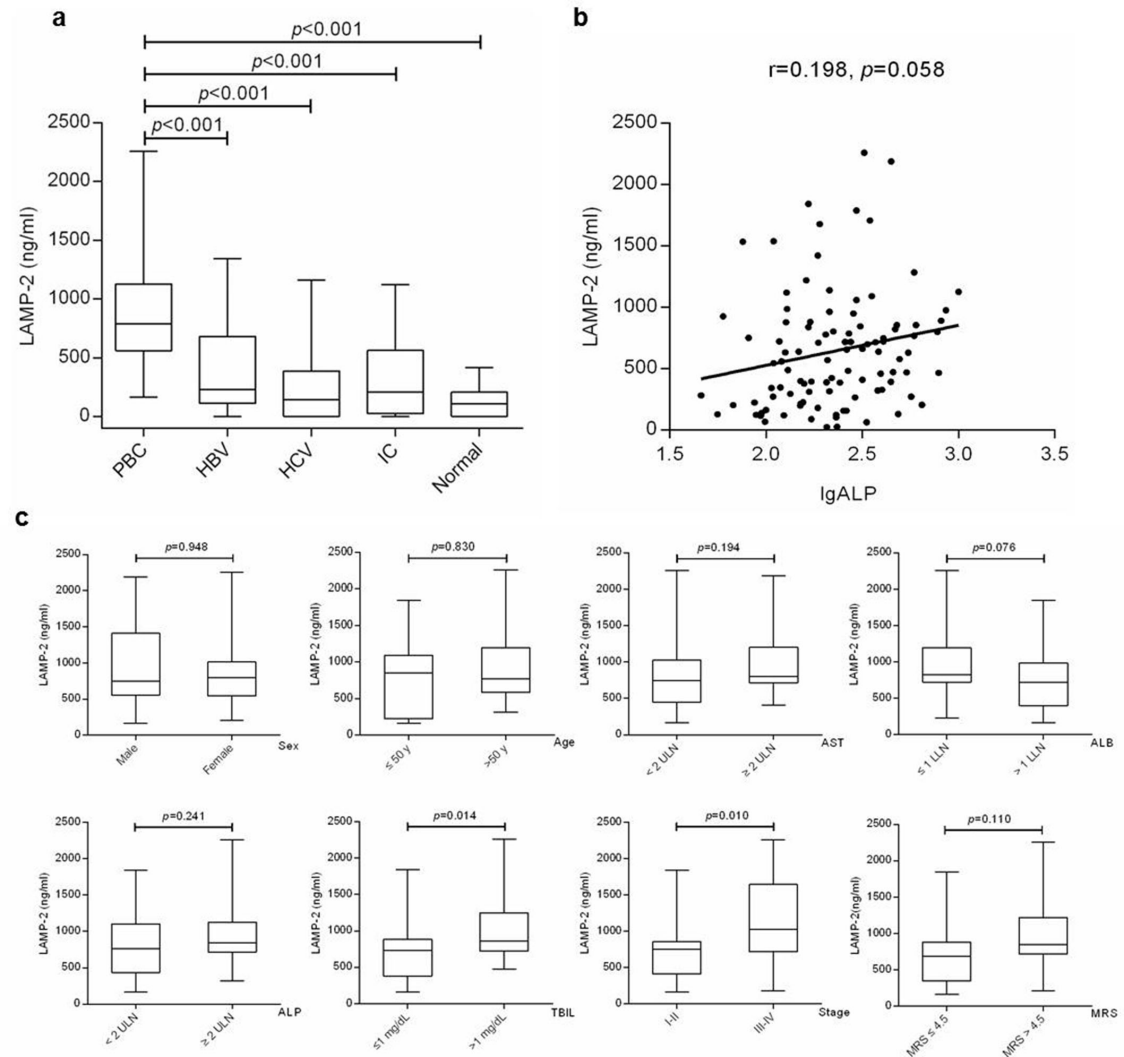
Baseline serum levels of LAMP-2 were increased in PBC, especially in patients with stage III-IV or TBIL > 1mg/dL. (a) Elevation of baseline serum LAMP-2 levels in patients with PBC. Sera of 102 PBC patients, 126 HBV patients, 114 HCV patients, 27 IC patients, and 84 healthy volunteers were evaluated for the serum LAMP-2 levels at baseline by ELISA. (b) Correlation between baseline LAMP-2 level and ALP activity. No significance correlation was found in patients with PBC between baseline LAMP-2 level and ALP activity (r = 0.198, *p* = 0.058). (c) Serum levels of LAMP-2 were increased in PBC patients with stage III-IV or TBIL > 1mg/dL. Data are expressed as median with interquartile range.

To determine the baseline serum levels of LAMP-2 in PBC, we measured the LAMP-2 in serum samples before treatment collected from PBC patients and controls by Enzyme-linked immunosorbent assay (ELISA) (Fig. 1a). The baseline serum LAMP-2 was significantly higher in patients with PBC (884.26 ng/mL in average, ranged from 559.12-1126.87), than those in patients with HBV (386.45 ng/mL in average, ranged from 112.07-681.55, *p*<0.001), HCV (245.33 ng/mL in average, ranged from 96.89-385.46, *p*<0.001), IC (302.26 ng/mL in average, ranged from 25.56-561.35, *p*<0.001), and healthy controls (128.33 ng/mL in average, ranged from 37.92-207.00, *p*<0.001). However, no significant difference in baseline serum LAMP-2 was observed among the control groups. We also divided patients as control into three clinical groups with different cirrhosis stages, hepatitis without cirrhosis, compensated cirrhosis and decompensated cirrhosis, to evaluate the effect of cirrhosis stage on baseline serum LAMP-2. Although there was a slight elevation of serum LAMP-2 in the decompensated cirrhosis group, no statistically significant difference among the three groups was found (Supplementary Figure 1). The elevation of baseline LAMP-2 in PBC patients may be accompanied by elevated level of ALP. However, no correlation was found between baseline serum LAMP-2 and ALP activity (r = 0.198, *p* = 0.058, Fig. 1b).

Given that PBC predominantly occurred in the middle-aged females, we tried to determine the influence of age and sex profile on the baseline serum LAMP-2 in PBC (Fig. 1c). Baseline serum LAMP-2 were slightly higher in male patients than that in female patients (945.00 ng/mL in average, ranged from 552.25-1410.63 versus 863.47 in average, ranged from 545.64-1012.67, *p* = 0.948) and in age > 50-year patients than in age < 50-year patients (928.37 ng/mL in average, ranged from 585.52-1196.63 versus 809.96 in average, ranged from 224.48-1091.39, *p* = 0.830), but these differences were statistically insignificant.

Previous studies have shown that PBC patients with ALP ≥ 2 ȕ upper limit of normal (ULN) and bilirubin (ALB) > 1 mg/dL were more likely to develop clinical endpoints^23^ and baseline ALB, advanced histologic stage and Mayo risk score (MRS) > 4.5 have been identified as the independent prognostic factors of PBC^9^,^24^. We then evaluated the baseline serum LAMP-2 in PBC patients grouped by these factors (Fig. 1c). Median serum LAMP-2 levels were slightly higher in AST ≥ 2 ULN group than in AST < 2 ULN group (998.32 ng/mL in average, ranged from 715.85-1205.20 versus 827.23 in average, ranged from 452.01-1024.44, *p* = 0.194), in ALB ≤ 1ȕ lower limit of normal (LLN) group than in ALB > 1 LLN group (1009.55 ng/mL in average, ranged from 721.20-1196.63 versus 772.89 ng/mL in average, ranged from 398.83-986.43, *p* = 0.076), in ALP ≥ 2ȕ ULN group than in ALP < 2ȕ ULN group (980.55 ng/mL in average, ranged from 714.91-1126.86 versus 805.16 ng/mL in average, ranged from 435.46-1100.45, *p* = 0.241), and in MRS > 4.5 group than in MRS ≤ 4.5 group (998.83 ng/mL in average, ranged from 675.74-1267.63 versus 744.77 ng/mL in average, ranged from 410.39-879.23, *p* = 0.078), but no significant differences were found. However, serum LAMP-2 was increased in late-stage group (Scheuer stages III and IV) compared with that in early-stage group (Scheuer stages I and II) (1131.95 ng/mL in average, ranged from 716.65-1643.10 versus 724.46 ng/mL in average, ranged from 410.39-854.51, *p* = 0.010) (Fig. 1c). This result suggested that high baseline serum LAMP-2 may indicate an advanced histological stage of PBC. Moreover, we found the median serum LAMP-2 was significantly elevated in total bilirubin concentration (TBIL) > 1 mg/dL group compared with TBIL ≤ 1 mg/dL group (1064.33 ng/mL in average, ranged from 721.42-1251.72 versus 758.20 ng/mL in average, ranged from 379.61-882.33, *p* = 0.014) (Fig. 1c).

### Baseline serum levels of LAMP-2 may not independently predict the response to UDCA treatment in patients with PBC

**Figure 2:**
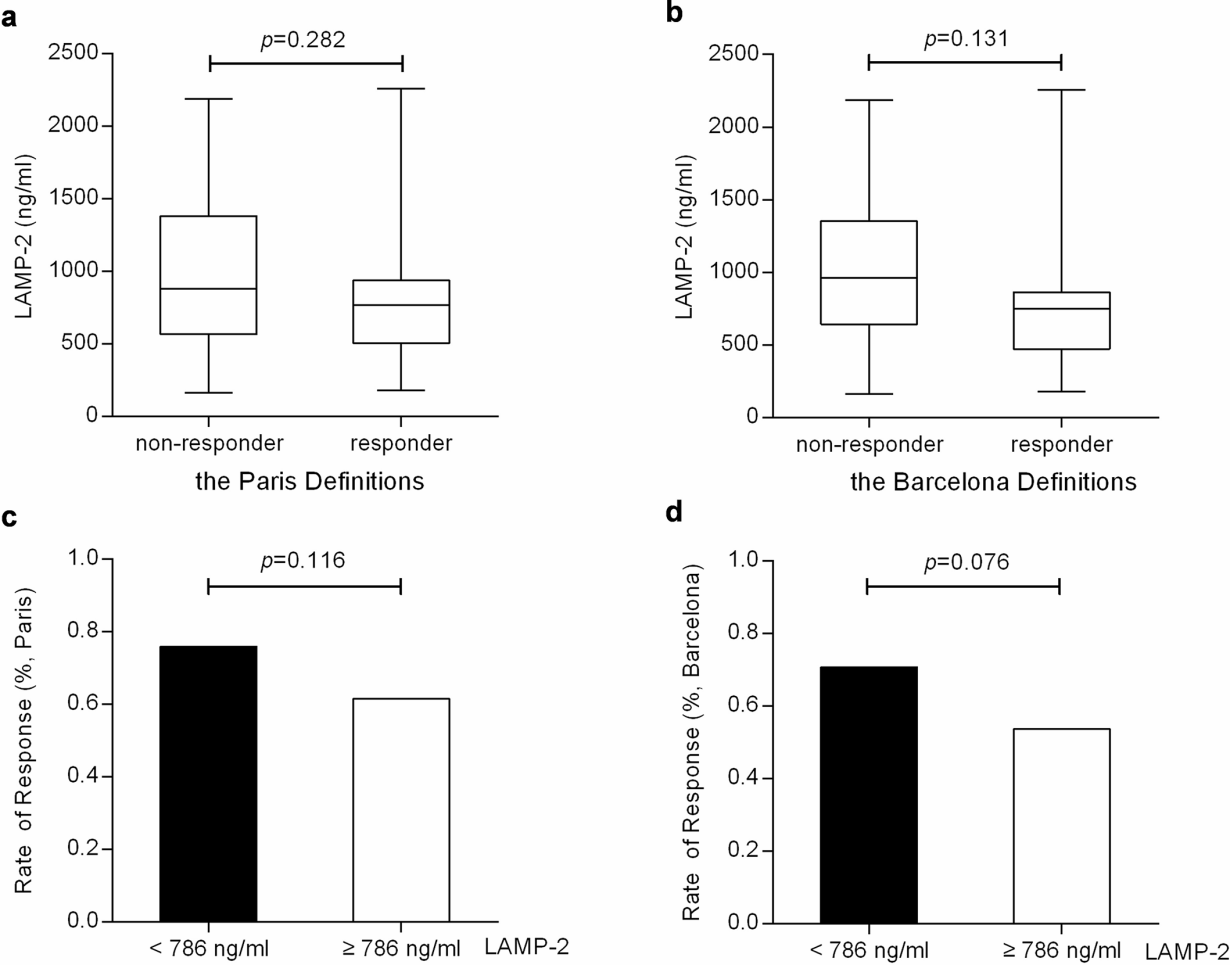
Baseline serum LAMP-2 levels in responders and non-responders and rates of biochemical response in low and high LAMP-2 groups. (a, b) The pretreatment serum LAMP-2 levels were higher in non-responders, but no significant differences were observed. (c, d) The rates of biochemical response were higher in low LAMP-2 group, but the differences were statistically insignificant. The biochemical responses were evaluated by Paris Definitions (a and c) or by Barcelona Definitions (b and d). Data are expressed as median with interquartile range in a and b.

To examine whether pretreatment serum LAMP-2 level could serve as a predictor of biochemical response to UDCA, we evaluated the baseline serum LAMP-2 in responders and non-responders. The Paris and Barcelona definitions of the 1-year biochemical response were independently utilized as criteria^7^,^9^. In our cohort, the rates of biochemical response were 68.6% (70/102) when defined by Paris criteria and 61.8% (63/102) by Barcelona criteria. The pretreatment levels of LAMP-2 were higher in non-responders than that in responders by Paris criteria (1023.14 ng/ml in average, ranged from 567.42-1382.21 versus 831.70 ng/ml in average, ranged from 507.55-939.03, *p* = 0.282) and by Barcelona criteria (996.77 ng/ml in average, ranged from 643.48-1352.56 versus 805.50 ng/ml in average, ranged from 471.45-864.14, *p* = 0.131), although no significant differences were observed (Figs. 2a and b). We then divided the patients with PBC into low (< 786 ng/ml) and high (≥ 786 ng/ml) LAMP-2 groups based on median pretreatment serum LAMP-2 level. The rates of biochemical response were 76.0% in low and 61.5% in high LAMP-2 group by Paris criteria (*p* = 0.116), while 70.8% in low and 57.3% in high LAMP-2 group by Barcelona criteria (*p* = 0.076) (Figs. 2c and d). These data indicated that pretreatment serum levels of LAMP-2 could not be exclusively and statistically associated with response to UDCA. The baseline serum LAMP-2 may not independently predict the response to UDCA treatment.

### Serum LAMP-2 level decreased during UDCA treatment in PBC patients

**Figure 3:**
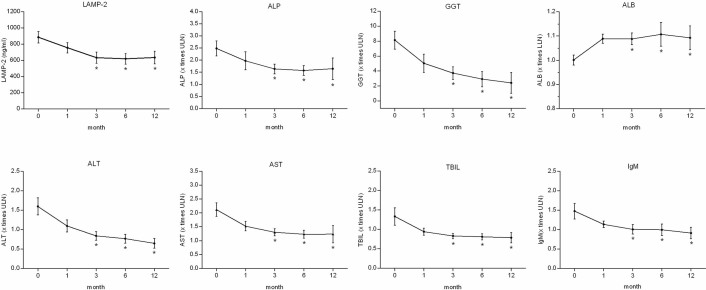
Serum LAMP-2 decreased over time for PBC treated with UDCA. Serum levels of LAMP-2 began to decline in the first month, and a more profound decline was found in the first three months, which was accompanied by a significant decrease in ALP, GGT, ALT, AST, TBIL and IgM, and elevation of ALB. Data are expressed as mean ± SEM. *p<0.001 versus baseline.

To clarify the relationship between serum LAMP-2 level and UDCA treatment, we determined the dynamics of serum LAMP-2 level, as well as ALP, gamma-glutamyl transferase (GGT), ALB, alanine aminotransferase (ALT), AST, TBIL, and immunoglobulin M (IgM) levels, within the first year of UDCA treatment (Fig. 3). Serum levels of LAMP-2 began to decline in the first month after treatment. A more profound decline was found in the first three months (*p*<0.001), which was accompanied by a significant decrease in ALP, GGT, AST, ALT, TBIL and IgM (*p*<0.001), and elevation of ALB (*p*<0.001). This indicated that an early decline of LAMP-2 as short as 3 to 6 months after UDCA treatment might be used as a predictor instead of traditional indicators introduced at 1 year after UDCA therapy.

### Serum LAMP-2 decreased more potently in responders to UDCA treatment

**Figure 4:**
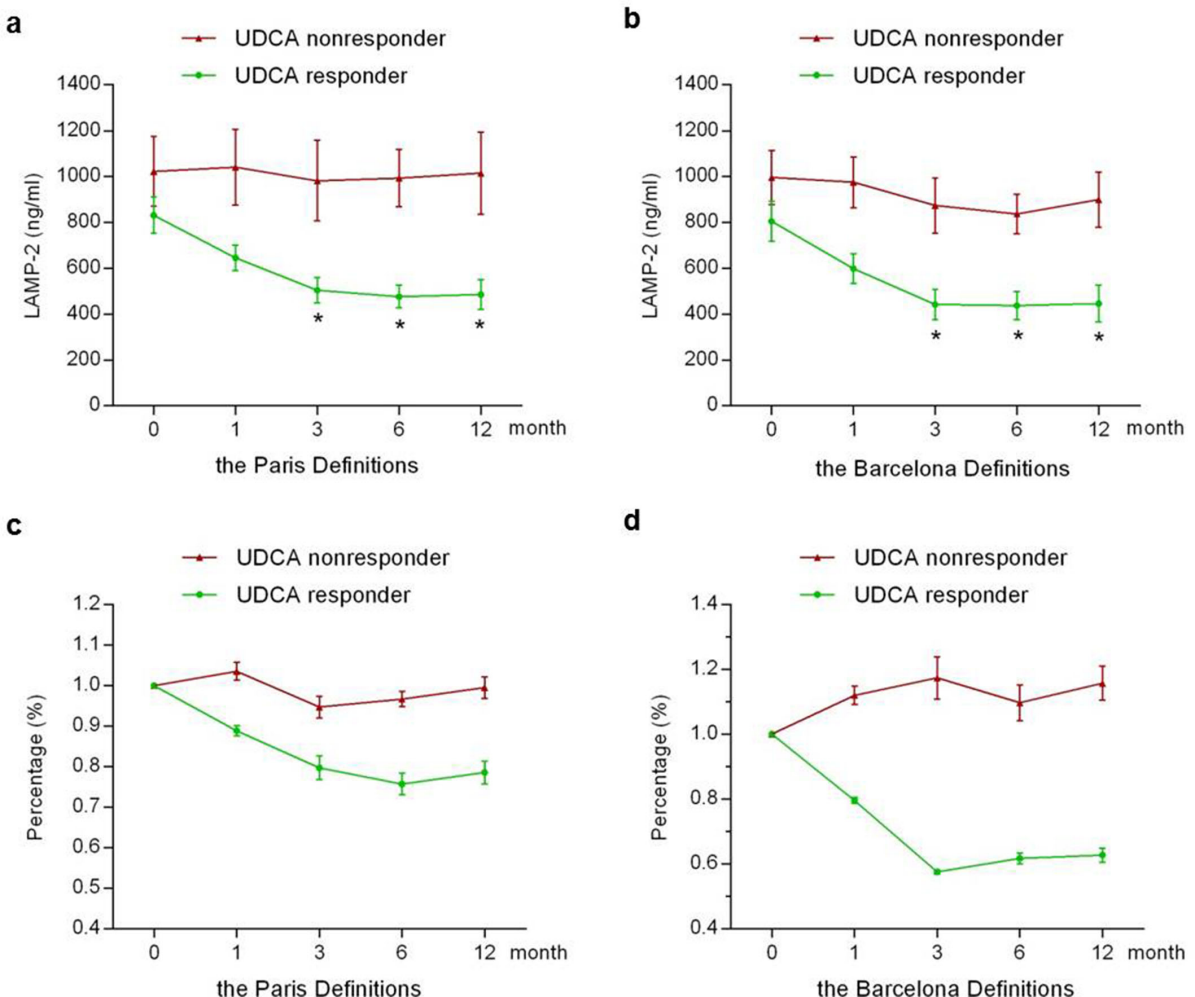
The changes of serum LAMP-2 levels in responders and non-responders after 1 year of UDCA treatment. (a, b) The prominent decline in LAMP-2 was noted in the third month in responders. (c, d) The changes of serum LAMP-2 levels in responders and non-responders. Each datum was normalized by dividing it by its corresponding baseline. The biochemical responses were evaluated by Paris Definitions (a and c) or by Barcelona Definitions (b and d). Data are expressed as mean SEM. *p<0.001 versus baseline.

Under UDCA therapy, a prominent decline in serum LAMP-2 was noted in the third month in responders, and gradually decreased subsequently, with a maximum decrease observed at the sixth month (Figs. 4a and b, green; *p*<0.001). However, serum LAMP-2 was decreased slightly in non-responders (Figs. 4a and b, red), even with an elevation at 1 year (Fig. 4b). After 1 year of UDCA treatment, serum LAMP-2 was decreased by about 42% with Paris criteria, and 45% with Barcelona criteria in responders, while in non-responders it was decreased by about 2% with Paris criteria and 10% with Barcelona criteria. The changes of serum LAMP-2 after 1 year of UDCA therapy are shown in Figs. 4c and d, in which each datum was normalized by dividing it by its corresponding baseline.

**Figure 5:**
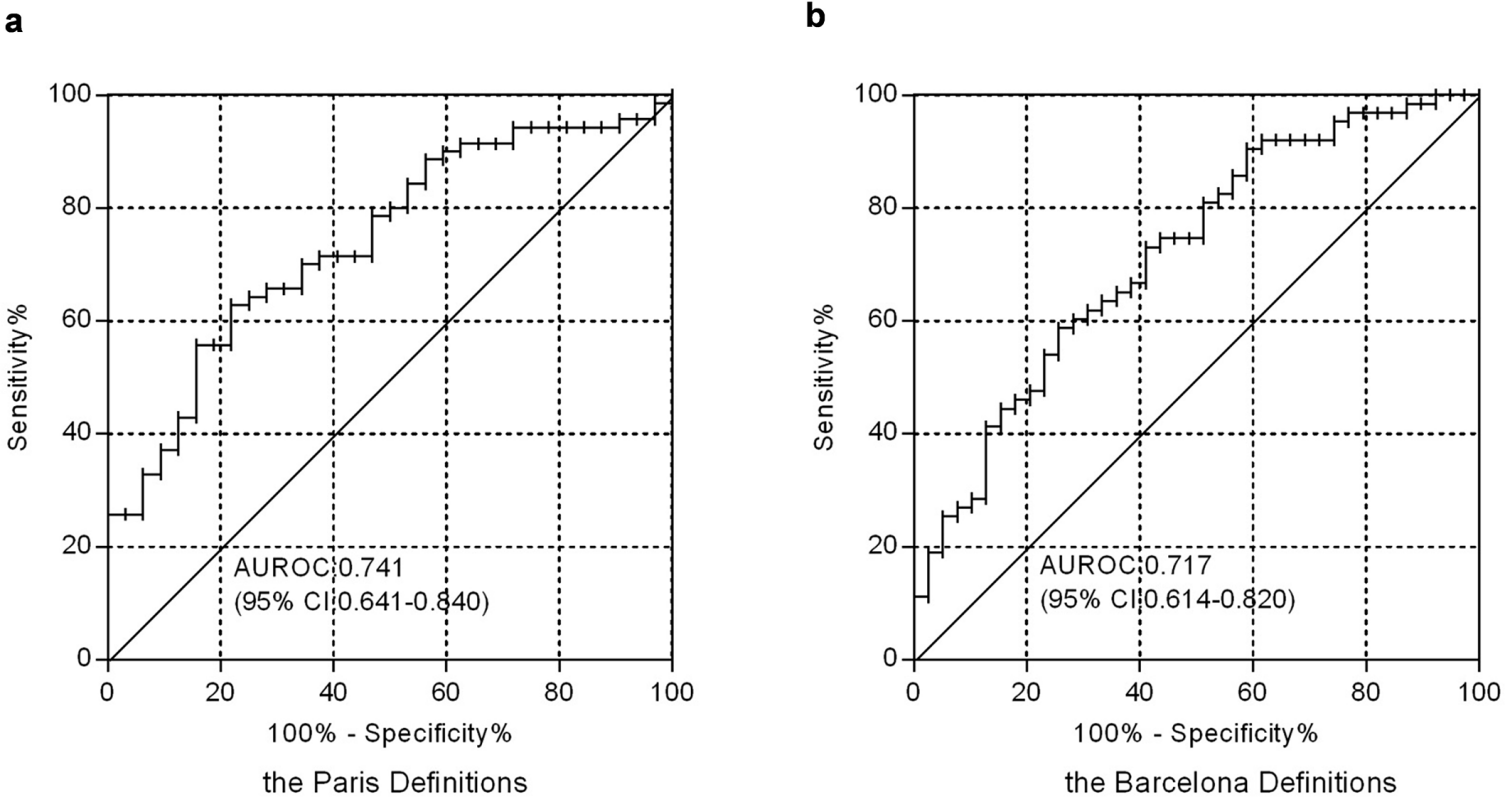
Clinical significance of LAMP-2 decrease for identifying biochemical response at 3 months of UDCA therapy. Among patients with PBC (n = 102), LAMP-2 decrease had an area under the ROC curve of 0.741 (95%CI: 0.641-0.840) by Paris criteria (a) or 0.717 (95%CI: 0.614-0.820) by Barcelona criteria (b) to identify biochemical response at 3 months of UDCA therapy in patients with PBC.

When LAMP-2 decline was used to identify biochemical response at 3 months of UDCA therapy among the 102 patients with PBC, the area under the ROC (Receiver Operating Characteristic) curve for LAMP-2 decline was 0.741 (95%CI: 0.641-0.840; Fig. 5a) by Paris criteria, or 0.717 (95%CI: 0.614-0.820; Fig. 5b) by Barcelona criteria. Furthermore, a decrease in LAMP-2 greater than 35% was observed with a predictability of 67% to response by Paris definitions, alongside a sensitivity of 62.9% and a specificity of 75.0%. While defined by Barcelona criteria, the predictability was 64%, alongside a sensitivity of 63.5% and a specificity of 64.1%. These results indicated that the 35% decline of LAMP-2 level after treatment for 3 months could be stated as an indicator of UDCA response.

### Validation of LAMP-2 dynamics to identify UDCA-responder

**Figure 6:**
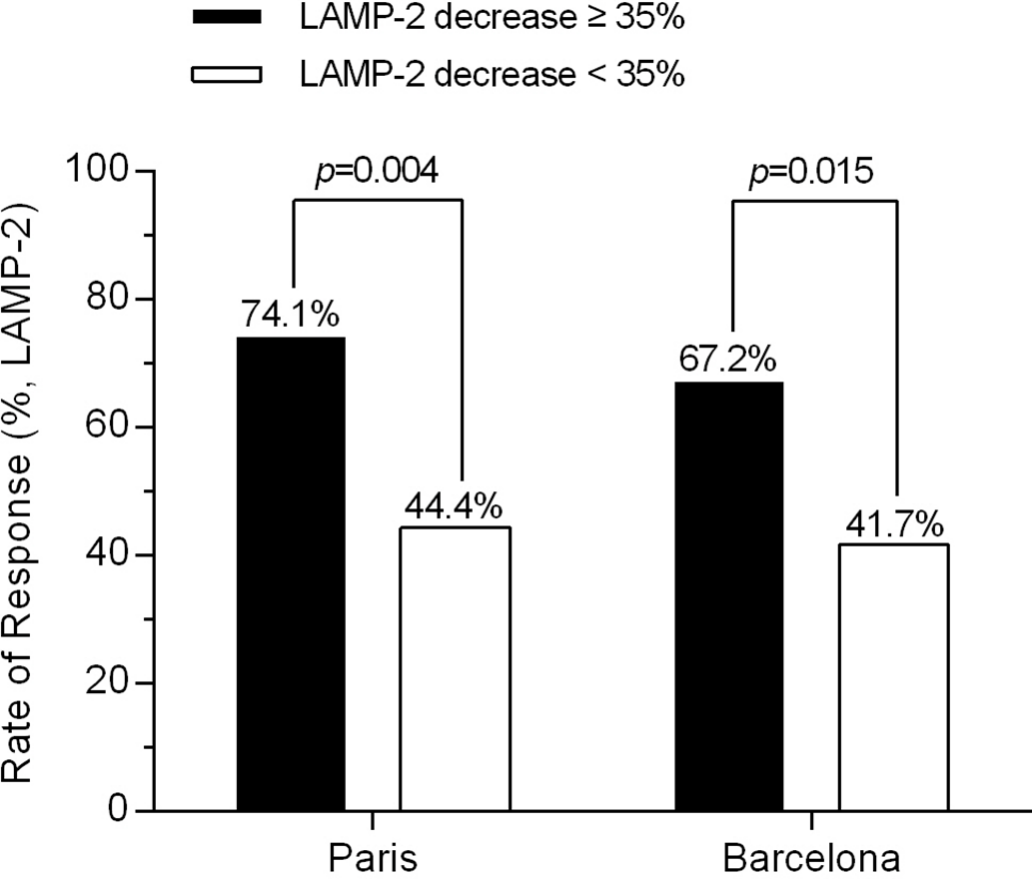
Validation of LAMP-2 decrease for identifying UDCA-responder at 3 months of treatment. In the validation cohort of 87 PBC patients, who experienced a LAMP-2 decline ≥ 35% after 3 months of UDCA have shown higher response rates both by Paris and Barcelona criteria.

A total of 87 consecutive PBC patients with available follow-up information and serum samples were enrolled in the validation cohort. The characteristics of the patients at enrollment and after 1 year of UDCA therapy were shown in Supplementary Table 1. At baseline, more than half of the patients (57%) were in an early stage of PBC. After 1 year of UDCA treatment, a total of 56 patients (responders, 64.4%) and 52 patients (responders, 59.8%) responded to UDCA according to Paris and Barcelona definitions, respectively. When we used the criteria of 35% LAMP-2 decline to identify UDCA-responder in this validation cohort, we found that the patients who experienced a LAMP-2 decline ≥ 35% after 3 months of UDCA treatment showed higher response rates both by Paris and Barcelona criteria (Figure 6). This was in accordance with our findings mentioned above in the retrospective investigation.

## Discussion

The biochemical response to UDCA is an independent predictive factor for death and liver transplantation^7^,^9^ and was recommended as one of the study endpoints in clinical trials where traditional endpoints were deemed unfeasible^14^. Thus, it is necessary and important to identify patients with incomplete biochemical response as soon as possible.

Herein, we aimed to determine the serum levels of LAMP-2 in patients with PBC and to evaluate whether serum levels of LAMP-2 could serve as a predictor of poor outcome after UDCA treatment in patients with PBC. Using a retrospective design we analyzed the data of 102 patients with a follow-up period of 1 year. The highlights of our investigation could be epitomized as that the serum levels of LAMP-2 in PBC patients prominently decreased within the first 3 months of UDCA therapy, and a 35% decline in serum LAMP-2 levels may help to identify the patients with satisfactory biochemical response.

When determining the pretreatment serum LAMP-2 levels in PBC, we found baseline serum LAMP-2 was significantly elevated in patients with PBC, compared with control groups, including HBV, HCV, IC and healthy volunteers. This increase in serum LAMP-2 in patients with PBC may be a phenomenon secondary to ALP, a surrogate marker of PBC. However, no correlation was found between serum LAMP-2 level and ALP activity. Several prognostic markers including biochemical and histological features have been shown to be predictive of the development of cirrhosis or liver failure^25^,^26^,^27^. We showed here that pretreatment serum LAMP-2 level was increased in late-stage patients with PBC (Fig. 1c), indicating a correlation between serum LAMP-2 and histological stage. Atbaseline, LAMP-2 levels were higher in non-responders, although the differences were not statistically significant (Figs. 2a and b). These results suggested that baseline serum LAMP-2 could beassociated with biochemical response, but it may not be adequate to predict the response to UDCA treatment independently.

Previously published criteria for predicting outcome of treatment were mainly based on biochemical response after 1 or 2 years of UDCA therapy^7^,^8^,^9^,^10^. However, it has been recommended that therapeutic trials should target patients with incomplete biochemical response after 3 to 6 months of UDCA treatment^14^. In our study, an obvious decline in serum LAMP-2 was found in the first three months (Fig. 3), which was accompanied by a significant decrease of ALP, GGT, AST, ALT, TBIL and IgM, and elevation of ALB in serum. This result suggests that the dynamics of LAMP-2 at 3 to 6 month of UDCA treatment may be used to identify patients with good or poor prognosis.

When evaluating the evolution of serum LAMP-2 within the first year of UDCA treatment in responders and non-responders, we observed a profound decline of LAMP-2 after 3 months of UDCA therapy especially in responders (Figs. 4a-d). A hypothesis that a decline in LAMP-2 after 3 months of UDCA therapy may be used to identify biochemical response to UDCA treatment was subsequently proposed. We found that a 35% decline had a sensitivity of 62.9% and a specificity of 75.0% in identifying cases of responders by Paris criteria, while a sensitivity of 63.5% and a specificity of 64.1% by Barcelona criteria. Therefore, we proposed that a 35% decline in LAMP-2 at 3 months after UDCA therapy might be used to prospectively identify the patients with satisfactory response to UDCA, which was further confirmed in the independent validation cohort.

In our retrospective cohort study, there were 61% patients with PBC were in the early stage, and 39% in the late stage, we then separately evaluated the decrease of LAMP-2 level to identify UDCA-responder at 3 months of treatment for patients based on their disease stages. We found that the LAMP-2 decrease ≥ 35% groups showed higher response rates in both early and late-stage PBC, but no significant differences were found in the early stage (Supplementary Figure 2). This might be attributed to a relatively slight increase of LAMP-2 in early-stage PBC patients and the insufficient scale of enrollment.

There were some limitations of our study, such as the small sample size and the retrospective design which was inevitably accompanied with potential biases including selection and recall bias. To avoid bias, we compared the basal characteristics of patients with PBC and controls. They did not differ statistically in age and sex (data not shown). The efficacy of LAMP-2 dynamics to evaluate long-term course of PBC, such as development of ascites, death, and need for transplantation, was not possible in this study. But these end point events and their relation with LAMP-2 could be worthy of future investigation. Our study may be a stepping stone to assessing the clinical utility of serum LAMP-2 level. The relatively low Area Under the ROC Curve (AUC) in our study, which seemed not a perfect criteria, might result from a lower proportion of cirrhosis at baseline in our study (10% versus 17%). However, since nowadays most patients are diagnosed and treated at an early stage, traditional hard end-points, such as the occurrence of death or liver transplantation, are no longer thought to be realistic^14^. We thus suggested that our approach might provide a good threshold for identification, particularly when used in conjunction with the Paris criteria and Barcelona criteria.

In human, LAMP-2 deficiency is the cause of Danon disease^20^,^28^ with cholesterol accumulation in patients. Further study reported that LAMP-2, its luminal domain in particular, plays a critical role in intracellular cholesterol transport^29^. In this study, we found the median serum LAMP-2 level was significantly elevated in TBIL > 1 mg/dL group (Fig. 1C), suggesting that there may be some kind of relation between serum LAMP-2 level and cholestatsis.

In summary, the present study suggested that high baseline serum levels of LAMP-2 might indicate an advanced histological stage of PBC and a 35% decrease in serum LAMP-2 might be used as a predictor to identify patients who would undergo response to UDCA therapy.

### Patients and methods

#### Patient Population

This study enrolled 102 patients with PBC who were newly diagnosed and followed up at Xijing Hospital (Xi'an, Shaanxi, China) between 2007 and 2013. Diagnosis of PBC was based on liver function tests, presence of serum antimitochondrial antibodies, and histopathological findings according to Ludwig's classification^30^. PBC patients were treated with UDCA at a daily dose of 13 to 15 mg/kg. Liver biochemical assay including serum bilirubin concentration, ALP, GGT, AST, ALT, and ALB as well as IgM levels were recorded at entry and repeated at 1, 3, 6, and 12 months during the first year of UDCA treatment. Ineligibility criteria included the use of corticosteroids or immunosuppressive drug in the preceding 6 months or features suggestive of other coexistent liver diseases including autoimmune hepatitis overlap syndrome, primary sclerosing cholangitis, alcoholic liver disease, Wilsons disease, a positive serology for hepatitis B virus or hepatitis C virus, and a follow-up of less than 1 year. Patients with complications of cirrhosis and those who underwent or were awaiting liver transplantation were also excluded.

The age- and gender-matched controls were grouped as follows: 126 subjects with viral hepatitis B, 114 subjects with viral hepatitis C, 27 subjects with intrahepatic cholestasis (including autoimmune hepatitis, primary sclerosing cholangitis, Gilbert's disease, Dubin-Johnson syndrome and drug induced cholestasis), and 84 healthy volunteers with histologically normal livers.

A validation cohort of 87 consecutive patients with PBC was enrolled at a different clinical site (Yongji Hospital of Liver Diseases, Shanxi, China) between 2005 and 2013 using the same exclusion and inclusion criteria to validate the decrease of LAMP-2 for identifying UDCA-responder at 3 months of treatment. This study was approved by the Ethics Committee of Xijing Hospital, and was carried out in accordance with the Declaration of Helsinki (2004). All patients had signed informed consent form.

### ELISA

Serum levels of LAMP-2 in enrolled populations were measured by ELISA kits (R&D Systems, Abingdon, UK) according to the manufacturers instruction. All the samples were assessed in duplicate. Plates were read using the Thermo Fisher (MA, USA) microplate reader and LAMP-2 concentration was calculated from the standard curve by the plate-reader software.

### Definitions of Biochemical Response

The biochemical response to UDCA was defined after 12 months of treatment according to two previously published definitions respectively: (1) Paris criteria (ie, ALP level ≤ 3 times ULN; AST level ≤ 2 times ULN, and normal bilirubin level)^31^, and (2) Barcelona criteria (ie, a decrease in ALP level > 40% of baseline level or a normal level)^32^.

### Statistical Analyses

All data were analyzed using SPSS v17.0 (SPSS, Inc., Chicago, IL, USA). KruskalWallis H test for multi-groups and MannWhitney U test for two groups were used to compare the difference for serum levels of LAMP-2. Correlation coefficient'ssignificance was assessed using Spearman's rank test. Comparisons between biochemical variables before and after 1, 3, 6, or 12 months of UDCA treatment were performed using the Wilcoxon signed-rank test for paired data. Differences in proportions for categorical variables were determined using Chi-square test. The sensitivity and specificity were calculated for a decline in serum LAMP-2 to identify biochemical response to UDCA. Where continuous data were distributed normally, they are presented as means standard deviation. Where data were distributed non-normally, they are presented as median and 25^th^-75^th^ interquartile range. All analysis were two-sided, and *P*<0.05 was considered statistically significant.

## Author Contributions

Conceived and designed the experiments: YH and YQS; Performed the experiments: LW and GYQ; Analyzed the data: LW, JBW, QY, XMZ, JWZ, XLR and DMF; Collected the samples and interpreted the data: YC, QL, ZYH, RRC and XZ; Wrote the paper: LW, GYQ, YH and YQS.

## Figures and Tables

**Table 1:** Biochemical characteristics of patients with PBC (n = 102) at baseline and after 1 year of UDCA treatment.

Characteristics	At Baseline	After 1 Year	* P Value
Female sex	76F, 26M		
Age (years)	53.54 ± 10.16		
ALT (IU/L)	53.68 ± 34.44	26.00 ± 11.49	*p* <0.001
AST (IU/L)	66.42 ± 42.27	43.20 ± 24.70	*p* <0.001
ALB (g/L)	39.47 ± 4.87	43.70 ± 4.41	*p* <0.001
TBIL (mg/dL)	1.41 ± 1.37	0.95 ± 0.36	*p* <0.001
ALP (IU/L)	291.34 ± 194.43	186.20 ± 133.18	*p* <0.001
GGT (IU/L)	309.08 ± 267.07	109.00 ± 140.59	*p* <0.001
IgM (g/L)	4.41 ± 2.84	2.73 ± 0.97	*p* <0.001
Histological stage (%)			
Early (I-II)	62 (61%)		
Late (III-IV)	40 (39%)		

Quantitative data are expressed as the mean SD.

*Wilcoxon signed-rank test for paired data.

Reference values: ALT, 7-40 IU/L; AST, 13-35 IU/L; ALB, 40-55 g/L; TBIL, 0.199-1.199 mg/dL; ALP, 50-135 IU/L; GGT, 7-45 IU/L; IgM, 0.5-3 g/L.

**Table 2:** Biochemical characteristics of the controls at baseline.

Characteristics	HBV	HCV	IC	Healthy Controls
Female sex	90F,36M	88F,26M	20F,7M	68F,16M
Age (years)	49.52 ± 12.82	48.30 ± 13.29	52.73 ± 11.05	53.02 ± 9.34
ALT (IU/L)	42.33 ± 49.08	80.38 ± 77.85	68.67 ± 127.74	18.37 ± 9.25
AST (IU/L)	40.82 ± 34.23	66.10 ± 45.42	83.41 ± 127.55	19.67 ± 8.45
ALB (g/L)	44.13 ± 5.35	40.28 ± 5.97	41.54 ± 5.84	53.23 ± 4.07
TBIL (mg/dL)	0.98 ± 0.67	1.03 ± 0.71	2.52 ± 3.01	0.62 ± 0.34
ALP (IU/L)	87.72 ± 63.32	92.38 ± 38.99	162.43 ± 157.35	73.85 ± 45.21
GGT (IU/L)	23.17 ± 30.80	59.79 ± 65.57	98.62 ± 237.75	48.91 ± 47.01
IgM (g/L)	-	-	-	-

Quantitative data are expressed as the mean SD.
